# Near-Peer Transition for Otolaryngology Emergency On-Call Practice: A Mixed-Methods Pilot Evaluation

**DOI:** 10.3390/healthcare14213786

**Published:** 2026-11-05

**Authors:** Surita Aodeng, Chuan Chen, Yang Zha, Xiaowei Wang, Wei Lv, Yizhen Wei

**Affiliations:** 1 Department of Otorhinolaryngology Head and Neck Surgery, Peking Union Medical College Hospital, Chinese Academy of Medical Sciences & Peking Union Medical College, Beijing 100730, China; aodengsurita@pumch.cn (S.A.);; 2 Department of Education, Peking Union Medical College Hospital, Chinese Academy of Medical Sciences & Peking Union Medical College, Beijing 100730, China

**Keywords:** near-peer teaching, workplace-based learning, graduate medical education, graded autonomy, transition to practice, otolaryngology, emergency care

## Abstract

Background/Objectives: Transitioning junior residents from supervised training to independent emergency on-call practice is a high-stakes stage of postgraduate workforce development. In otolaryngology, this requires rapid clinical judgment, procedural competence, and timely escalation. We evaluated a workplace-embedded Near-Peer Transition Program (NPTP) designed to support progression toward independent emergency duty. Methods: We conducted a single-center, uncontrolled convergent mixed-methods pilot evaluation. The NPTP combined pre-duty workshops with a one-month transition phase in which 10 junior residents were paired with five senior residents during emergency on-call shifts, progressing from observation to assisted and supervised performance. Evaluation included paired junior-resident pre–post surveys, senior-resident and faculty surveys, retrospective faculty readiness ratings, and semi-structured interviews. Results: Twenty participants completed the evaluation (10 junior residents, five senior residents, and five faculty members). Junior residents’ self-rated overall preparedness increased from 3.0 ± 1.15 to 3.8 ± 0.92 on a five-point scale. Exploratory item-level analyses suggested higher perceived readiness in several clinical domains. Senior residents reported perceived improvements in junior performance and benefits to their own teaching and leadership, with low perceived additional workload. Faculty reported favorable perceptions of safety and feasibility; 86% of individual faculty readiness ratings were ≥4 on a five-point scale. Qualitative feedback highlighted progressive confidence building, an enhanced safety net, and reciprocal learning. Conclusions: This small pilot supports the feasibility and perceived educational value of the workplace-embedded NPTP. Graded near-peer supervision may provide a practical workforce development strategy bridging simulation-based preparation and autonomous practice in high-acuity settings. Larger comparative studies are needed to establish effectiveness.

## 1. Introduction

Otolaryngology emergencies, including severe epistaxis, acute upper airway obstruction, and deep neck infections, require timely assessment, procedural competence, and appropriate escalation under considerable clinical pressure [1,2]. For junior residents, the transition from supervised training to independent emergency on-call practice therefore represents a particularly demanding stage of professional development. Although residents may have completed formal teaching and simulation-based preparation, readiness for independent practice can remain variable, and this transition is frequently accompanied by uncertainty and stress [3,4]. Supporting residents as they assume increasing clinical responsibility is consequently an important component of postgraduate workforce development, particularly in high-acuity settings where trainee autonomy must be balanced with appropriate supervision and patient safety [5].

Conventional preparation for emergency duty typically combines faculty-led teaching, simulation, case-based learning, and supervised clinical exposure. These approaches are valuable for developing foundational knowledge and procedural skills, and simulation provides a controlled environment in which learners can rehearse uncommon or high-risk scenarios [6]. However, structured educational activities cannot fully reproduce the unpredictability, time pressure, competing clinical demands, and responsibility associated with authentic emergency care. A gap may therefore persist between competence demonstrated in a controlled educational setting and readiness to act with greater autonomy in routine clinical practice. Competency-based medical education emphasizes progressive responsibility and the need to support transitions toward independent practice through observation and supervision in authentic clinical environments [7]. Workplace-based transitional models may therefore complement conventional teaching and simulation by allowing residents to develop autonomy while retaining timely access to supervision and escalation.

Near-peer teaching (NPT), in which a trainee at a more advanced stage provides teaching or supervision to a less experienced colleague, represents one potential approach to such workplace-based transition support [8]. Its educational value has been explained partly through cognitive congruence, whereby near peers may be able to explain recently acquired knowledge and practical reasoning in ways that are accessible to junior learners, and social congruence, whereby reduced hierarchical distance may facilitate communication and help-seeking [9]. These characteristics may be particularly relevant during emergency on-call transitions, when junior residents must make time-sensitive decisions but may also need to disclose uncertainty, seek clarification, or escalate care. A psychologically supportive supervisory relationship may facilitate these behaviors while allowing responsibility to increase progressively [9,10].

Near-peer approaches have previously been used in otolaryngology education, including teaching modules and acute ENT workshops, while simulation-based boot camps have also been developed to improve preparedness for common clinical and procedural challenges [11,12,13]. However, much of the published literature has focused on undergraduate learners, discrete teaching sessions, or simulation-based training rather than structured near-peer supervision embedded within routine emergency clinical work. Consequently, less is known about how near-peer supervision may support junior residents during the specific transition from supervised preparation to independent on-call responsibility. To address this gap, we developed a structured Near-Peer Transition Program (NPTP) that incorporated a one-month period of graded near-peer supervision into routine otolaryngology emergency workflows. This mixed-methods pilot study aimed to evaluate the program’s feasibility, perceived educational value and changes in junior residents’ self-reported readiness for independent emergency duty, senior residents’ experiences as near-peer supervisors, and faculty perceptions of safety and readiness for transition to independent practice.

## 2. Materials and Methods

### 2.1. Study Design and Participants

We conducted a single-center, uncontrolled convergent mixed-methods pilot program evaluation in the Department of Otolaryngology–Head and Neck Surgery at Peking Union Medical College Hospital, a tertiary academic teaching hospital in Beijing, China. The Near-Peer Transition Program (NPTP) was embedded within the department’s routine residency training and emergency on-call preparation. The program evaluation was conducted between August 2025 and October 2025.

Participants comprised 10 junior residents (JRs), five senior residents (SRs), and five faculty members (FACs). JRs were postgraduate year (PGY)-1 or PGY-2 residents who had not yet begun independent otolaryngology emergency on-call duty at the start of the transition period. SRs had prior independent otolaryngology emergency experience and resident teaching responsibilities. FACs were attending physicians responsible for second-line supervision of emergency care and residency training. All JRs completed both pre- and post-transition evaluations. Participation in the research evaluation was voluntary and did not affect residents’ formal training or assessment.

### 2.2. Near-Peer Transition Program

The NPTP was integrated into the existing emergency rotation workflow without requiring additional clinical shifts and comprised three sequential phases.

First, pre-duty preparation included lectures, simulation workshops, and case-based discussions covering common and high-risk otolaryngology emergencies, including epistaxis management, airway emergencies, abscess drainage, clinical prioritization, and departmental escalation pathways.

Second, during the one-month transition phase, each JR shadowed SRs for at least four emergency night shifts, approximately one per week. JR–SR pairings varied across shifts. SRs received no program-specific preceptor training but had previously completed the shadowing phase themselves. JRs progressed from observation to assisted participation and procedural performance under direct SR observation throughout the procedure. SRs provided immediate guidance and sought faculty assistance when further support was required. No standardized supervision checklist or formal competency framework was used. Escalations to faculty were not prospectively logged for this evaluation.

Third, after completing the transition phase, JRs began independent emergency on-call duty, with faculty backup remaining available according to standard departmental escalation procedures. The NPTP was implemented uniformly for residents entering emergency on-call responsibilities during the study period.

### 2.3. Measures and Data Collection

Three locally developed questionnaires were used to evaluate the program from the perspectives of JRs, SRs, and FACs. Item development was informed by the near-peer teaching literature, residency transition frameworks, and the departmental emergency training curriculum. Draft questionnaires were reviewed by three otolaryngology faculty members with medical education experience for relevance and clarity using a four-point rating scale. Items with an item-level content validity index (I-CVI) below 0.80 were revised or removed. Two residents who were not included in the study subsequently pilot-tested the questionnaires for clarity and comprehensibility. These locally developed instruments have limited psychometric validation; questionnaire findings should therefore be interpreted as preliminary.

The JR questionnaire comprised 12 closed-ended items, administered immediately before (T0) and after (T1) the one-month transition phase, together with three open-ended questions. Closed-ended items assessed self-perceived readiness, procedural confidence, communication, stress, and overall preparedness for independent emergency duty. Items were rated on a five-point Likert scale from 1 (strongly disagree) to 5 (strongly agree). Eleven closed-ended items had identical wording at T0 and T1. The anxiety item assessed anticipated-duty anxiety at T0 and perceived anxiety reduction at T1; these were not treated as equivalent repeated measures. The open-ended questions also differed between time points.

The SR questionnaire comprised nine closed-ended items addressing perceived junior development, teaching and feedback experience, leadership, workload, and satisfaction, together with three open-ended questions. Closed-ended items used a five-point Likert scale from 1 (strongly disagree) to 5 (strongly agree). The workload item was negatively worded, with lower raw scores indicating less perceived additional workload. The FAC questionnaire comprised one resident-specific global readiness item, ten program-level closed-ended items, and three open-ended questions. Each of the five FACs retrospectively rated all 10 JRs individually, recalling their readiness when they first began independent emergency duty. The global readiness item served as the Faculty Global Assessment of Junior Readiness (F-GAJR) and was rated from 1 (not ready for independent duty) to 5 (fully independent and safe). Ratings of 4 or 5 were considered indicative of faculty-perceived readiness for independent emergency duty. The F-GAJR was used as a pragmatic descriptive measure rather than a validated entrustment instrument. The program-level items assessed perceived educational benefits, patient safety, faculty workload, feasibility, sustainability, satisfaction, and potential transferability, using a scale from 1 (strongly disagree) to 5 (strongly agree).

Qualitative data were obtained from open-ended questionnaire responses and semi-structured interviews with all 20 participants (10 JRs, five SRs, and five FACs). Each participant completed one interview lasting approximately 10 min. Interviews explored experiences of supervision, perceived learning gains, confidence, workload, safety, and transition to independent duty. The complete questionnaires are provided in Supplementary File S1.

### 2.4. Data Analysis

Quantitative analyses were performed using Python 3.12.14 and SciPy 1.17.0. Questionnaire ratings: Continuous variables were summarized using means and standard deviations, and categorical variables using counts and percentages where appropriate. Changes between T0 and T1 were evaluated for nine identically worded JR readiness/confidence items using Wilcoxon signed-rank tests with exact permutation *p*-values. Mean paired differences were also reported with approximate t-based 95% confidence intervals. No overall questionnaire score was analyzed. Stress-relief, satisfaction, and the differently worded anxiety items were summarized descriptively.

SR and FAC questionnaire results were summarized descriptively. Statistical tests were two-sided. All item-level comparisons were exploratory because of the small sample size and multiple comparisons. Neither *p*-values nor confidence intervals were adjusted for multiple comparisons.

Open-ended questionnaire responses and interview feedback were reviewed together and summarized descriptively to identify recurring views on the program. No formal coding framework or dedicated qualitative software was used. Independent double-coding was not undertaken; therefore, a coder-disagreement resolution procedure was not applicable. Qualitative feedback was considered alongside the quantitative findings during interpretation and presented in a joint display (Table 1).

### 2.5. Ethical Considerations

The study was conducted in accordance with the Declaration of Helsinki. The Medical Research Ethics Review Committee of Peking Union Medical College Hospital determined that the study was exempt from full ethics review No.I–26ZM0069 because it evaluated an existing educational practice embedded within routine residency training, used de-identified data, and posed minimal risk to participants. Written informed consent for research participation and use of de-identified questionnaire and interview data was obtained from all participants. Participation was voluntary, and data were de-identified before analysis.

## 3. Results

### 3.1. Participants

Twenty participants completed the program evaluation, including 10 junior residents (JRs), five senior residents (SRs), and five faculty members (FACs). All JRs completed both the pre- and post-transition questionnaires.

### 3.2. Junior Resident Outcomes

Mean self-rated overall preparedness for independent emergency duty increased from 3.0 ± 1.15 at T0 to 3.8 ± 0.92 at T1 (mean paired difference 0.80; unadjusted 95% CI 0.50–1.10). Exploratory item-level analyses also indicated higher self-reported ratings for independent emergency assessment, prioritization and escalation, anterior nasal packing, posterior nasal packing or escalation, peritonsillar abscess drainage, airway crisis management, and clinical handover. Communication ratings showed a smaller change (unadjusted *p* = 0.188). Stress-relief, satisfaction, and the differently worded anxiety items were summarized descriptively (Table 1).

Qualitative questionnaire feedback illustrated perceived gains in confidence. One JR wrote, “Performing procedures myself after a demonstration can greatly boost my confidence” (JR01, T1 questionnaire; translated excerpt).

### 3.3. Senior Resident and Faculty Evaluations

SRs reported favorable perceptions of junior residents’ development during the transition phase, including triage and escalation (4.2 ± 0.84) and procedural skills (4.4 ± 0.89). They also reported benefits to their own clinical reasoning (4.6 ± 0.55) and communication, feedback, and leadership (a combined item, 4.4 ± 0.89). Perceived additional workload was low (1.8 ± 0.84), and overall satisfaction was 4.0 ± 1.00 (Table 2). Workload was not objectively measured.

Faculty reported favorable perceptions of reduced patient risk (4.8 ± 0.45), feasibility (4.8 ± 0.45), sustainability (4.8 ± 0.45), and overall satisfaction (4.4 ± 0.55). Across the five faculty members’ retrospective assessments of ten JRs, 43 of 50 individual F-GAJR ratings (86%) were ≥4 (Table 3). This percentage refers to ratings, not residents; it reflects perceived readiness rather than demonstrated competence or measured patient safety.

### 3.4. Qualitative Feedback and Integration

Descriptive review of qualitative feedback highlighted three recurring areas. Progressive confidence building reflected JRs’ gradual movement from anxiety and observation toward greater participation and perceived autonomy. One JR nevertheless described increased apprehension after becoming more aware of the urgency of some clinical conditions. An enhanced safety net described the value participants placed on immediate near-peer support while maintaining access to faculty escalation. Reciprocal learning reflected perceived benefits for both learner and supervisor: JRs gained practical guidance in communication, prioritization, and emergency management, whereas SRs described development in teaching, feedback, and leadership.

Table 4 links quantitative findings with related qualitative feedback. Higher readiness ratings and accounts of supervised participation provide complementary support for perceived educational value. Low SR-reported workload coexisted with JR concerns about limited teaching during busy shifts. Favorable faculty perceptions therefore do not establish uniform implementation or objective safety benefits.

**Table 4:** Joint display of quantitative findings and qualitative questionnaire feedback.

Aspect	Quantitative Finding	Related Feedback	Representative Questionnaire Excerpt	Integrated Interpretation
Readiness	Overall preparedness: 3.0 ± 1.15 to 3.8 ± 0.92 (JR, *n* = 10).	Confidence building	“Performing procedures myself after a demonstration can greatly boost my confidence” (JR01).	Higher self-ratings and supervised-practice accounts support perceived readiness; competence was not tested.
Reciprocal learning	Own clinical reasoning: 4.6 ± 0.55; communication, feedback, and leadership: 4.4 ± 0.89 (SR, *n* = 5).	Learning through teaching	“Working with them also helps me revisit the fundamentals of procedures” (SR03).	Both sources describe educational benefits for supervisors.
Workload and teaching	Additional workload: 1.8 ± 0.84 (SR, *n* = 5; lower indicates less burden).	Variable teaching availability	“Sometimes, when things get too busy, there is no time for teaching” (JR08).	Low reported burden coexisted with constrained teaching during busy shifts.
Support and feasibility	Perceived risk reduction and feasibility: each 4.8 ± 0.45 (FAC, *n* = 5).	Team support	“In the emergency department, I am not alone; the whole emergency team is there” (JR07).	These are complementary perceptions of support and feasibility, not measured safety outcomes.

Values are mean ± sample SD. Quotations are translated excerpts from open-ended questionnaire responses; JR quotations are from T1. Participant identifiers are anonymized. Overall preparedness refers to a single five-point item.

## 4. Discussion

This mixed-methods pilot evaluation examined a structured near-peer transition program embedded within routine otolaryngology emergency practice. The principal finding was that junior residents reported greater readiness for independent emergency duty after completing the one-month transition phase, with improvements observed across several clinically relevant areas including emergency assessment, prioritization, selected procedures, airway crisis recognition, and handover. Senior residents perceived benefits not only in junior development but also in their own teaching, feedback, and leadership, while reporting little additional workload. Faculty evaluations were favorable regarding program feasibility and perceived safety, and most individual retrospective faculty readiness ratings were ≥4. Qualitative findings complemented these observations by identifying progressive confidence building, an enhanced safety net, and reciprocal learning as central features of the transition experience. Taken together, these findings suggest that structured near-peer supervision may provide a feasible bridge between formal preparation and increasing autonomy in high-acuity workplace settings.

The principal contribution of the NPTP lies in its focus on the transition from educational preparation to real clinical responsibility. Previous near-peer initiatives in otolaryngology have commonly involved undergraduate learners or discrete educational activities, including supplementary teaching modules and acute ENT workshops [11,12]. Simulation-based boot camps similarly provide structured exposure to procedural and clinical scenarios before independent practice [13]. These approaches are valuable for developing knowledge and technical confidence but occur primarily within controlled educational environments. In contrast, the NPTP incorporated graded supervision directly into routine emergency on-call work, allowing junior residents to encounter authentic uncertainty, competing clinical demands, and escalation decisions while retaining immediate access to a more experienced near peer. The present findings therefore extend previous otolaryngology education studies by examining near-peer support as a workplace-based transition strategy rather than as a stand-alone teaching intervention.

Several mechanisms may explain why this model was perceived as useful. Near-peer support may facilitate communication and help-seeking through cognitive and social congruence [9,10,14,15], while graded participation is consistent with situated learning [16,17]. These mechanisms may help explain the themes of confidence building and an enhanced safety net. Importantly, the present study does not demonstrate objective improvement in competence or patient safety; rather, the convergence of self-reported readiness, supervisor perceptions, and qualitative accounts suggests that the NPTP may create a supportive environment for graded participation in real clinical work.

The program also has potential relevance to workforce development and competency-based postgraduate training. Contemporary competency-based medical education emphasizes progression toward independent practice based on observed performance and increasing entrustment rather than time in training alone [7,18,19]. A structured transition period may provide additional opportunities for direct workplace observation before junior residents assume independent on-call responsibility. In the present study, faculty global readiness ratings were used only as pragmatic retrospective judgments and should not be interpreted as validated entrustment decisions. Nevertheless, the model illustrates how near-peer supervision could complement faculty oversight by providing more frequent observation, immediate feedback, and progressive responsibility within routine service delivery. The low additional workload reported by senior residents is also relevant to implementation, because educational interventions that depend heavily on additional faculty time may be difficult to sustain in busy clinical departments. Future studies should align readiness judgments with specialty-specific entrustable professional activities and prospective workplace-based assessments incorporating direct observation [20,21].

Several limitations should be considered. First, this single-center pilot included 10 junior residents, five senior residents, and five faculty members; the paired analysis therefore involved only 10 residents, limiting statistical power and precision and reducing confidence in the stability and generalizability of the findings. Without a comparison group, the observed changes cannot be attributed solely to the NPTP. All item-level comparisons and their confidence intervals were exploratory and unadjusted for multiple comparisons; individual *p*-values should not be interpreted as confirmatory evidence of effectiveness. Second, the principal outcomes were based on self-reported readiness, supervisor perceptions, and retrospective global ratings. Although a T0 self-report baseline was available, no objective baseline assessment of competence was obtained. Faculty global ratings were not aligned item by item with the JR questionnaire and were not an objective reference standard; therefore, the study could not quantify the discrepancy between perceived readiness and demonstrated competence. Objective workplace-based assessments, escalation patterns, procedural outcomes, adverse events, and other patient-level safety indicators were not systematically available, precluding conclusions regarding actual clinical competence or patient safety. Third, the questionnaires were locally developed and, although subjected to content review and pilot testing, require further validation in larger cohorts. Fourth, qualitative findings were derived from a relatively small group within a single institutional culture and may not capture experiences in other training systems. Finally, workload was perceived rather than objectively measured, and costs, resource use, and cross-specialty implementation were not evaluated; scalability therefore remains to be established. Future multicenter studies should evaluate the NPTP in larger and more diverse residency programs, incorporate prospective workplace-based assessments and patient-level outcomes, and examine whether transition duration should be individualized according to demonstrated readiness. These steps would help determine whether structured near-peer transition can provide a scalable approach to supporting safe progression toward independent practice across high-acuity specialties.

## 5. Conclusions

This small mixed-methods pilot evaluation provides preliminary evidence of the feasibility and perceived educational value of a workplace-embedded Near-Peer Transition Program for otolaryngology emergency duty. Junior residents reported greater readiness, while senior residents and faculty described favorable educational and implementation experiences. Graded near-peer supervision may provide a practical bridge between simulation-based preparation and autonomous practice. Larger comparative studies incorporating workplace-based assessments and objective clinical outcomes are needed to determine effectiveness and transferability.

## Figures and Tables

**Table 1:** Junior residents’ self-reported questionnaire ratings before and after the transition phase.

Item	T0 Mean ± SD	T1 Mean ± SD	Mean Change (95% CI)	*p* Value
Confidence in independent emergency assessment	2.7 ± 1.06	3.8 ± 0.63	1.10 (0.57 to 1.63)	0.008
Prioritizing life-threatening problems and timely escalation	3.2 ± 1.14	4.2 ± 0.42	1.00 (0.25 to 1.75)	0.031
Readiness for anterior nasal packing	2.9 ± 1.37	4.1 ± 0.74	1.20 (0.54 to 1.86)	0.008
Posterior nasal packing or timely escalation	2.2 ± 1.14	3.2 ± 1.32	1.00 (0.66 to 1.34)	0.004
Peritonsillar abscess incision and drainage	2.0 ± 1.05	3.0 ± 1.33	1.00 (0.52 to 1.48)	0.008
Recognizing and initiating management of airway emergencies	3.1 ± 0.99	4.2 ± 0.63	1.10 (0.47 to 1.73)	0.008
Communication with nurses, anesthesiology, and colleagues	3.5 ± 0.85	4.0 ± 0.94	0.50 (−0.11 to 1.11)	0.188
Clinical handover	3.3 ± 1.16	4.5 ± 0.71	1.20 (0.54 to 1.86)	0.008
Overall preparedness for independent emergency duty	3.0 ± 1.15	3.8 ± 0.92	0.80 (0.50 to 1.10)	0.008
Teaching-related stress relief—descriptive	4.1 ± 1.10	4.3 ± 0.67	—	—
Satisfaction with shadowing—descriptive	3.8 ± 0.79	4.1 ± 0.88	—	—
Anxiety about forthcoming duty—T0 question	4.3 ± 1.06	—	—	—
Perceived anxiety reduction—T1 question	—	3.5 ± 1.18	—	—

Abbreviations: T0, pre-transition; T1, post-transition; SD, standard deviation. Individual items were rated on a five-point Likert scale. Item-level comparisons were exploratory and were not adjusted for multiple testing.

**Table 2:** Senior residents’ evaluations of the Near-Peer Transition Program.

Item	Mean ± SD
Perceived junior improvement in triage and escalation	4.2 ± 0.84
Perceived junior improvement in procedural skills	4.4 ± 0.89
Adaptability of teaching to junior level and emergency context	3.8 ± 1.30
Improvement in own clinical reasoning	4.6 ± 0.55
Improvement in communication, feedback, and leadership	4.4 ± 0.89
Confidence in emergency work as a preceptor	4.6 ± 0.55
Additional teaching workload	1.8 ± 0.84
Balance between teaching and emergency tasks	4.0 ± 1.22
Overall satisfaction	4.0 ± 1.00

Values are mean ± sample SD for five SRs. Items used a five-point agreement scale. Lower scores on the additional-workload item indicate less perceived additional workload. Communication, feedback, and leadership were assessed as one combined item.

**Table 3:** Faculty program evaluations and retrospective individual readiness ratings.

Measure	Result
Perceived improvement in junior triage and initial management	4.8 ± 0.45
Perceived improvement in timely and appropriate escalation	4.6 ± 0.55
Perceived improvement in overall readiness	4.6 ± 0.55
Perceived safety of decisions and procedures during shadowing	4.4 ± 0.55
Perceived reduction in patient risk	4.8 ± 0.45
No substantial additional faculty burden	4.8 ± 0.45
Program feasibility	4.8 ± 0.45
Program sustainability	4.8 ± 0.45
Overall satisfaction	4.4 ± 0.55
Perceived transferability	4.6 ± 0.55
Individual retrospective F-GAJR ratings ≥ 4	43/50 (86%)

Program evaluation scores are presented as mean ± sample SD for five faculty respondents, using five-point Likert scales. The F-GAJR ranged from 1 (not ready) to 5 (fully ready for independent duty). Each faculty member rated all 10 JRs, yielding 50 individual ratings. These repeated ratings were summarized descriptively; the percentage refers to ratings ≥ 4, not residents. F-GAJR, Faculty Global Assessment of Junior Readiness; JR, junior resident.

## Data Availability

The de-identified quantitative data supporting the findings of this study are available from the corresponding author upon reasonable request. Individual qualitative feedback is not publicly available because it may contain contextual information that could compromise participant confidentiality.

## Supplementary Materials

The following supporting information can be downloaded at: https://www.mdpi.com/article/10.3390/healthcare14213786/s1, Supplementary File S1: Questionnaires used in the Near-Peer Transition Program; Section S1: Junior Resident Self-Assessment Questionnaire; Section S2: Senior Resident Preceptor Questionnaire; Section S3: Faculty Evaluation Questionnaire.
