# Supplementary material for: Near-Peer Transition for Otolaryngology Emergency On-Call Practice: A Mixed-Methods Pilot Evaluation
Source: Healthcare (Basel). 2026 Nov 5;14(21):3786. doi: 10.3390/healthcare14213786 (PMC13762624; doi:10.3390/healthcare14213786)
Supplement: Supplementary file 1 [file healthcare-14-03786-s001.zip › healthcare-4564186-supplementary.pdf]

Supplementary File S1. Questionnaires Used in the Near-Peer Transition Program

Section S1. Junior Resident Self-Assessment Questionnaire

This questionnaire was administered to junior residents before and after the emergency shadowing intervention to assess self-perceived preparedness and confidence for independent emergency duty. Items 1–12 are rated on a 5-point Likert scale (1 = strongly disagree, 5 = strongly agree). Items 1–10 and 12 were identical at T0 (before) and T1 (after); item 11 and the open-ended questions differed as indicated below.

1. I feel confident in independently assessing patients presenting with otolaryngology emergencies.
2. I am able to identify life-threatening conditions and escalate care appropriately.
3. I am prepared to perform anterior nasal packing.
4. I am able to manage posterior nasal packing or seek timely senior support when required.
5. I am able to perform incision and drainage of a peritonsillar abscess.
6. I can recognise and initiate management of airway emergencies.
7. I can communicate effectively with emergency nurses, anaesthesiologists, and colleagues.
8. I am able to provide clear and structured clinical handovers.
9. Participation in the emergency shadowing programme reduced my stress related to independent duty.
10. Overall, I am satisfied with the emergency shadowing teaching model.
11. T0: I feel anxious about commencing independent emergency duty.
T1: My anxiety about emergency duty has decreased.
12. Overall, I feel adequately prepared to undertake independent emergency duty.

Open-ended questions at T0:

13. What are your main concerns about commencing independent emergency duty, and why?
14. What are the three most important competency gaps you hope to address during the shadowing period?
15. What types of support or guidance do you most expect from senior residents or faculty during emergency shadowing?

Open-ended questions at T1:

13. What was the greatest improvement you gained from the emergency shadowing period? Please give one specific example.
14. How have your anxiety or feelings of uncertainty changed, and what situations or support helped relieve them?
15. If you could improve one aspect of emergency shadowing, what would you choose? Please give an actionable suggestion.

Section S2. Senior Resident Preceptor Questionnaire

This questionnaire explored senior residents' perceptions of the educational value, feasibility, and impact of the near-peer transition program.

Background question: Years of independent ENT emergency duty: __________

Items 1–9 are rated on a 5-point Likert scale (1 = strongly disagree, 5 = strongly agree).

1. I was able to clearly observe improvements in junior residents' emergency triage and escalation skills.
2. I observed improvements in junior residents' procedural skills during the shadowing period.
3. I was able to adapt my teaching approach according to the junior resident's level and the clinical context.
4. Serving as a preceptor enhanced my own clinical reasoning during emergency duty.
5. Serving as a preceptor improved my communication, feedback, and leadership skills.
6. Participation as a preceptor increased my confidence during emergency duty.
7. The emergency shadowing programme increased my clinical workload.
8. I was able to balance teaching responsibilities with routine emergency clinical duties.
9. Overall, I am satisfied with the emergency shadowing teaching model.

Open-ended questions:

10. What benefits, if any, did you personally gain from participating as a preceptor?
11. In which areas did junior residents demonstrate the greatest improvement during the shadowing period?
12. If you could improve one aspect of the emergency shadowing model, what would it be?

Section S3. Faculty Evaluation Questionnaire

Faculty members retrospectively evaluated junior residents' readiness for independent otolaryngology emergency duty following completion of the near-peer transition program. Each of the five faculty members rated all ten junior residents individually, recalling readiness when they first began independent duty. Item 1 was completed once per resident; items 2–11 addressed the programme overall.

Items 1–11 are rated on a 5-point scale. Item 1 uses: 1 = not ready at all; 2 = almost unready, requiring intensive supervision; 3 = basically able, but requiring frequent guidance; 4 = ready for independent duty with minimal support; 5 = fully independent and safe. Items 2–11 use 1 = strongly disagree and 5 = strongly agree.

1. Overall, the resident was ready to undertake independent otolaryngology emergency duty.
2. Through emergency shadowing, junior residents' abilities in emergency triage and initial management have clearly improved.
3. Through emergency shadowing, junior residents can report to senior clinicians more promptly and appropriately in emergencies.
4. Through emergency shadowing, junior residents' overall readiness for independent duty has clearly improved.
5. Residents' clinical decision-making and procedures were performed within a safe and appropriate range.
6. The emergency shadowing model reduced potential risks to patient safety.
7. Implementation of the emergency shadowing model did not substantially increase faculty workload.
8. The emergency shadowing model was feasible within our department.
9. The emergency shadowing model is sustainable within routine clinical practice.
10. Overall, I am satisfied with the emergency shadowing teaching model.
11. This emergency shadowing model has potential value for dissemination to other training programmes.

Open-ended questions:

12. How did the emergency shadowing model influence residents' readiness for independent emergency duty?
13. Do you believe the emergency shadowing model supports resident development while ensuring patient safety? Please provide an example if possible.
14. If one aspect of the emergency shadowing model could be improved, what would you suggest?
